# How Is the Caregiver Doing? Capturing Caregivers’ Experiences With a Reflective Toolkit

**DOI:** 10.2196/13688

**Published:** 2019-05-28

**Authors:** Lilian Bosch, Marije Kanis, Julia Dunn, Kearsley A Stewart, Ben Kröse

**Affiliations:** 1 Amsterdam University of Applied Sciences Amsterdam Netherlands; 2 Duke University Durham, NC United States

**Keywords:** human-centered design, informal caregivers, experience sampling, home care, positive psychology, mental health, reflective technologies, well-being, HCI (human computer interaction)

## Abstract

**Background:**

This paper describes the *Co-Care-KIT*, a reflective toolkit designed to provide insights into the diverse experiences of home-based informal caregivers during the delivery of care to a relative or loved one.

**Objective:**

The aim of this study was to evaluate the toolkit, including a custom-designed journal, tools for photography-based experience sampling, and heart rate tracking, which enables caregivers to collect and reflect on their positive and negative daily experiences *in situ*.

**Methods:**

A 2-week field study with informal caregivers (N=7) was conducted to evaluate the Co-Care-KIT and to capture their daily personal emotional experiences. The collected data samples were analyzed and used for collaborative dialogue between the researcher and caregiver.

**Results:**

The results suggest that the toolkit (1) increased caregivers’ awareness of their own well-being through *in situ* reflection on their experiences; (2) empowered caregivers to share their identities and experiences as a caregiver within their social networks; (3) enabled the capturing of particularly positive experiences; and (4) provided caregivers reassurance with regards to their own mental health.

**Conclusion:**

By enabling capturing and collaborative reflection, the kit helped to gain a new understanding of caregivers’ day-to-day needs and emotional experiences.

## Introduction

Longer life expectancies and health care reforms in professional health care provision have increased the reliance on informal caregivers (eg, family, friends, or neighbors), who carry out long-term in-home care of their relative or loved one [1,2]. Across the Organization for Economic Co-operation and Development countries, such as France, the United Kingdom, the United States, and the Netherlands, informal caregivers have become the backbone of long-term care provision. Over 1 in 10 adults provide informal (unpaid) care to their parent (36%), spouse (31%), friend (18%), or relative (18%) [2]. Providing care can impact caregivers’ health and well-being in numerous ways. Understanding the diverse needs of informal caregivers is critical for finding ways for supporting their well-being. This is not only beneficial for them but also for the care recipients who often prefer someone they know well to provide (long-term) care for them [2]. Formal care providers should also have an interest in improving the well-being of informal caregivers, as informal caregivers play an imperative role in providing higher quality home-based care [3].

Technology has considerable potential to support home care provision. A large body of research exists in human-computer interaction (HCI) to mainly support patients [4-7] but also caregivers [6,8-13] in the context of in-home care. Thus far, however, in developing technologies for caregivers, research has generally emphasized functional support for caregiving (how can a caregiver provide better, more effective care?), whereas few studies focus on the well-being of caregivers [8,14,15]. Yet, researchers in HCI have already urged for moving and evolving from an efficiency approach toward a focus on user experience and toward finally embracing positive psychology approaches and focusing on wellness [16-20]. Similarly, in the development of technologies for home care, researchers in this field have already indicated the need for shifting focus from solutions for managing care to solutions for wellness [21,22]. The demand for and reliance on informal caregivers is likely to increase even more. Thus, it is now time to also acknowledge the remaining gap in technological approaches and solutions for positively identifying and supporting the caregiver experience and focus on how technological methods could place more importance on caregivers’ well-being.

In 2013, the National Alliance for Caregiving [14] already stated in a timely report how technology can play a more meaningful role in helping informal caregivers:

There is an ongoing need for research on family caregivers, especially as technology dramatically impacts caregiving. More current, thorough and accurate data is needed about the diversity of caregiver roles and responsibilities, about what caregiving involves day-to-day and the nature of the burden it represents, and how much it impacts those around the caregiver.

Better identifying and understanding the varying, complex, and personal experiences of caregivers in the contexts in which it takes place is crucial in exploring how (technological) solutions could improve the needs and well-being of caregivers. To address this, this study investigated how methods from HCI and design can help capture and understand the day-to-day (emotional) experiences of caregivers. For this purpose, a reflective study toolkit was designed. The Co-Care-KIT allows for caregivers to collect meaningful data about the personal, everyday experience of caregiving. The Co-Care-KIT serves 2 goals. First, to investigate how different tools for capturing caregivers’ experience combined can work during a 2-week field study. Second, to gain in-depth knowledge about the experience of diverse caregivers through the qualitative data that were collected with the toolkit. The primary purpose was thus to evaluate a toolkit for better understanding the experiences of caregivers. This was done so to meet the ultimate ambition of better supporting caregivers’ well-being. Consequently, by encouraging self-reflection, the use of the toolkit was designed to also be of value for the caregiver. A positive design approach was undertaken in trying to maximize the toolkit’s use for the participants by facilitating caregivers to collect and reflect on experiences that were meaningful to them as these occurred in the wild.

The rest of this paper presents the design of the Co-Care-KIT toolkit based on studies of relevant methods from HCI and design for capturing experiences. It describes a 2-week field study with the Co-Care-KIT that was conducted with home-based informal caregivers in diverse care situations (N=7). Then it reports the study’s findings, including an evaluation of the toolkit method. The paper concludes with a discussion of the lessons learned using this method. The main contributions of this paper constitute the design and evaluation of the rich toolkit; the lessons learned and new perspectives gained through deploying this reflective toolkit method; and findings concerning the emotional experience of caregivers from the rich data that were collected through this study. These findings build on and contribute to the research on how HCI can better understand and support informal caregiving.

### Capturing Experiences

#### Informal Caregiving Research

In investigating the experience of caregivers, research has focused primarily on the more negative aspects. It has focused particularly on the burden that caregivers endure, such as psychological stress; depression; poor health; guilt about not doing enough; work and employment problems; and social effects such as isolation [23-28]. Nonetheless, caring for a loved one can also provide a sense of gratification to caregivers. The lack of attention paid to the positive aspects of caregiving has skewed perceptions of the experience of caregivers in research [29]. However, there are also positive aspects that have been highlighted in the literature [28-32]. Recently, scholars have started to emphasize on how positive aspects of caregiving can actually lower levels of reported depression of caregivers [31]. Conceptualizations of positive aspects of caregiving include the following: role satisfaction (a sense of satisfaction in performing tasks); emotional rewards (feeling appreciated or successful); personal growth (growing as a result of the role); competence and mastery (sense of mastery in the new role); relationship gains; sense of duty; and reciprocity (satisfaction in fulfilling a sense of duty) [31]. Nonetheless, every care situation is unique and different factors can contribute to the experience of caregivers. For example, the care load of an informal caregiver often depends on the constantly changing needs of their care recipient. It also depends on the presence (or absence) of other informal and formal caregivers. Moreover, whether the helping role of a caregiver causes them stress or a feeling of satisfaction can quickly change based on time and context [33]. Studies have predominantly used quantitative measures to investigate the experience of caregivers. These do not capture the contextual features that determine the experience of a caregiver [23]. As the experience of caregiving is not a fixed state but a dynamic process that depends on many factors, it is crucial to recognize these nuances to be able to support the well-being of caregivers. Thus, in investigating the experience of caregivers, dynamic factors such as time and context should be considered in an ongoing assessment of how a caregiver is doing from moment to moment and day to day. Moreover, caregivers may not be aware of or focused on their own health and well-being, as previous research has shown that caregivers prioritize the well-being of the person they care for over their own [8,34]. Thus, in studying the experience of caregivers, it is important to also encourage caregivers to reflect on their personal well-being and needs and provide tools to do so. In the next section, this paper explores how methods from HCI and design can help to capture and understand the day-to-day experiences of caregivers.

## Methods

### Experience Research in Human-Computer Interaction and Design

In HCI, there are a number of methods that involve capturing, measuring, and tracking daily habits and experiences. For example, Quantified Self technologies, such as sensing and lifelogging systems (the automatic capture of activities with digital tools such as wearables), have demonstrated potential in collecting bodily and day-to-day status data of people. Although there are some notable exceptions, such as the Three Good Things method that encourages people to record positive experiences [16,35], lifelogging and sensing activities often seem to focus on detecting the more negative emotional, functional, and bodily experiences, such as anxiety, stress, and depression [36-40]. However, these technologies can also be deployed for more positive purposes. For example, Sellen and Whittaker [41] have discussed how lifelogging could potentially support learning through later reminiscence (recollection of past events) and reflection (reflecting on and reviewing of past events). In psychology research, reflection is increasingly correlated with mental health and well-being [42,43]. Consequently, in HCI studies, there is also a growing interest in design for reflection [35,44-48], often involving participants to self-track or capture data. For example, Isaacs et al [35] have developed a lifelogging app that facilitates users to record and reflect on memories, ultimately aiming to increase the well-being of its users. Moreover, diary studies [49] are now more widely used in HCI to evoke reflections on participants’ views and experiences *in situ.* In addition, the expression of emotions through expressive writing is a common therapy for improving a person’s well-being [50-52]. Furthermore, cultural probes [53] are materials used in design research to elicit expression and responses from users. For example, in a notable study in 2016 with informal caregivers, probes (in the form of diaries and photo cameras) were used as tools to gather stimulus for interviews [54]. Probes can come in many forms, such as diaries, postcards, cameras, objects, or games, and are typically combined in toolkits to enable rich and varied qualitative inquiry from prospective users [55]. Diary methods have been used in research to investigate social, psychological, and physiological processes within daily situations, and avoid some of the recall biases inherent to single report interviews [56]. Interpretation of data gathered through design probes is often mostly done by designers and researchers to ultimately inspire the (co-)design process of new products or services. Thus, using probes for collaborative interpretation and self-reflection by the target group has largely been uncharted territory so far. Another method that is specifically developed for measuring the experience of people is the *Experience Sampling Method* (ESM). ESM [57] originates from the field of positive psychology that places focus on the positive aspects of life and where it is commonly used for assessing *in situ* subjective happiness of people by having them report on their thoughts and feelings at signaled moments throughout the day [58]. It combines the ecological validity of naturalistic behavioral observation with elements of a diary and the precision of questionnaire measures [57]. This approach has also further been developed for the use with mobile devices and various media, such as photography, to capture data and experiences from users [59-61]. Photo elicitation allows for the development of rich phenomenological data to research the lived experience and provides subjects an opportunity to express their experience from their own perspective [62]. The process has been described as highly empowering because it offers an opportunity for research participants to actively engage in the research process [63]. ESM has an advantage over other research methods that also collect information about the context and lives of people (such as observations, interviews, or diary studies), namely, it enables direct capturing of experiences in the moment they occur. The Atlas of Caregiving [14] also stresses the need for experiential and reflective approaches toward the daily practices of caregiving but focuses on a different cultural (the United States) and social context (family).

### Design for Reflection

Baumer [64] coined the term *reflective informatics* and distinguishes between 3 dimensions (perspectives) to inform and inspire how design can potentially support reflecting: first, there is *breakdown* (eg, when a system suddenly does not perform as a user expects it to, an opportunity for reflection is created); second is *inquiry* (supporting reviewing of past experiences such as with reflecting on past journal entries); and the final dimension is *transformation* (not just examining the current state of something but envisioning alternatives and supporting change).

As this paper focuses on capturing and understanding the personal and varying daily experience of caregivers (or inquiring into the experience of caregiving), this study fits mostly into Baumer’s dimension of *inquiry*. We address this by enabling caregivers to self-capture experiences that are meaningful to them and, in doing so, facilitate and empower caregivers to reflect on their current and past experiences.

This study investigated methods from HCI, (reflective) design, and (positive) psychology for capturing the experience of people. Such methods have not yet been studied vastly with caregivers. Moreover, these methods focus on capturing samples by participants but often leave the interpretation of data to the researchers. Therefore, these investigations formed the basis for the design of a reflective study toolkit for caregivers: the Co-Care-KIT. This study kit contains a variety of tools that combined enable caregivers to capture and reflect on their varying, contextual (emotional), and personal experiences as these occur *in situ*. The tools not only enable capturing of (positive) experiences but make it possible to visualize these. In this way, the caregivers’ experiences can be discussed in a dialogue between the researchers and caregivers. The next section describes the design and development process of the Co-Care-KIT.

### Co-Care-Kit Design

**Figure 1:**
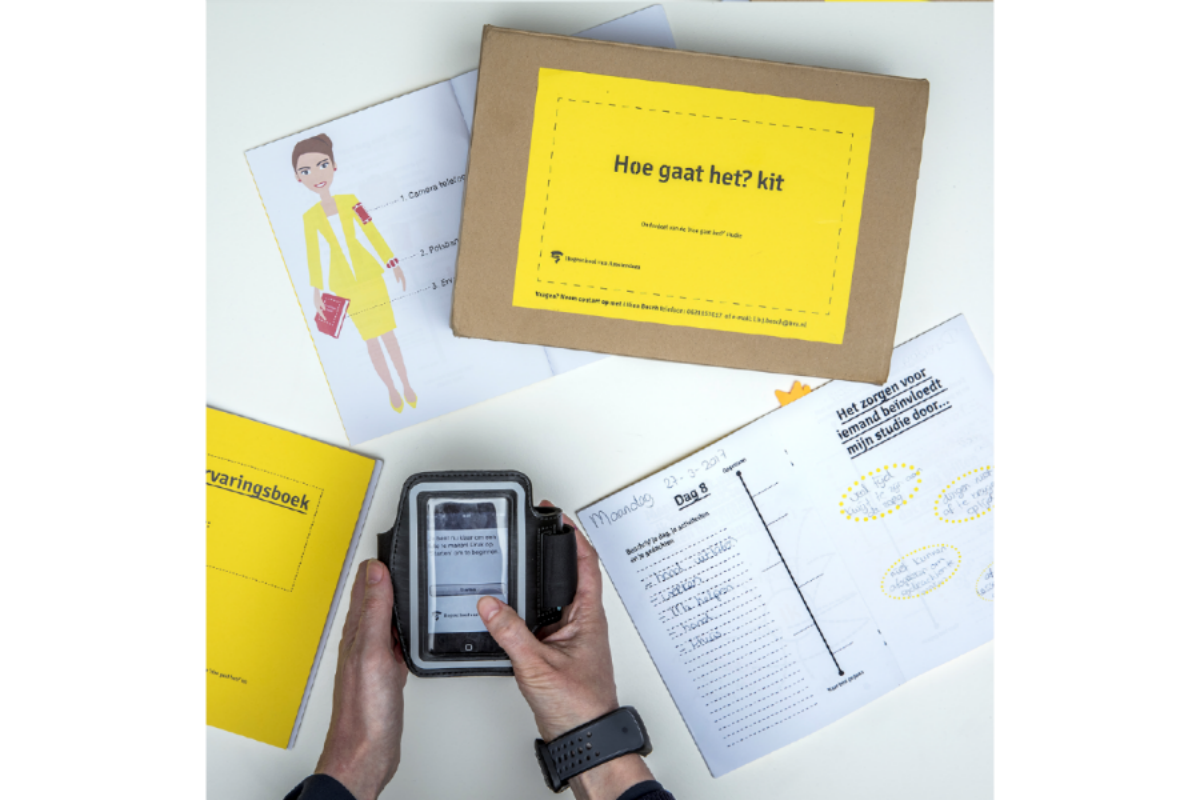
The Co-Care-KIT, including a custom-designed journal, photography-based experience sampling app, and heart rate tracker.

The idea of a toolkit for caregivers was also inspired by a study by Bosch and Kanis [8], which identified several design opportunities for caregivers. This study also highlighted how capturing and cherishing moments during caregiving could emphasize the caregivers’ positive outlook. Therefore, in the design of the individual tools in the Co-Care-KIT, emphasis was placed on eliciting reflections about how the caregiver was doing both in the moment and generally. A variety of complementary methods of elicitation were included to understand the caregiver experience fully. The final Co-Care-KIT toolkit (see Figure 1) comprises the following tools:

1. *A custom-designed journal* for writing and reflecting about daily experiences. In addition to recording daily thoughts and experiences, the journal contained predesigned pages for supporting experience disclosure.
2. *An iPhone with an app for photography-based experience sampling:* this app enabled caregivers to visually capture their experiences and rate how they are feeling at signaled moments throughout the day.
3. *A heart rate tracker wristband* that caregivers can use to continuously measure their heart rate variability (HRV), as an indication of their stress.
4. *Supplementary material:* Online and offline informative resources, such as bookmarks and leaflets, on caregiving support were included in the kit. Finally, additional writing aids to use with the notebook were provided, such as pens, markers, and Post-it notes.

A basic and unpolished look was chosen for the visual design of the Co-Care-KIT, using materials such as cardboard and paper, to maximize its appeal to a wide variety of participants (both young and adult caregivers, male and female) and to attract participants in using the provided tools and materials. These tools were selected because these could complement each other, namely, appealing to different types of users and ways of (positively) identifying the mental and experiential state. A key goal was that the caregivers could directly benefit from participation. The kit, therefore, not only provided additional supplements but also provided tools that enabled self-reflection so as to support more awareness of their own needs and well-being.

### Journal: Writing and Reflecting

**Figure 2:**
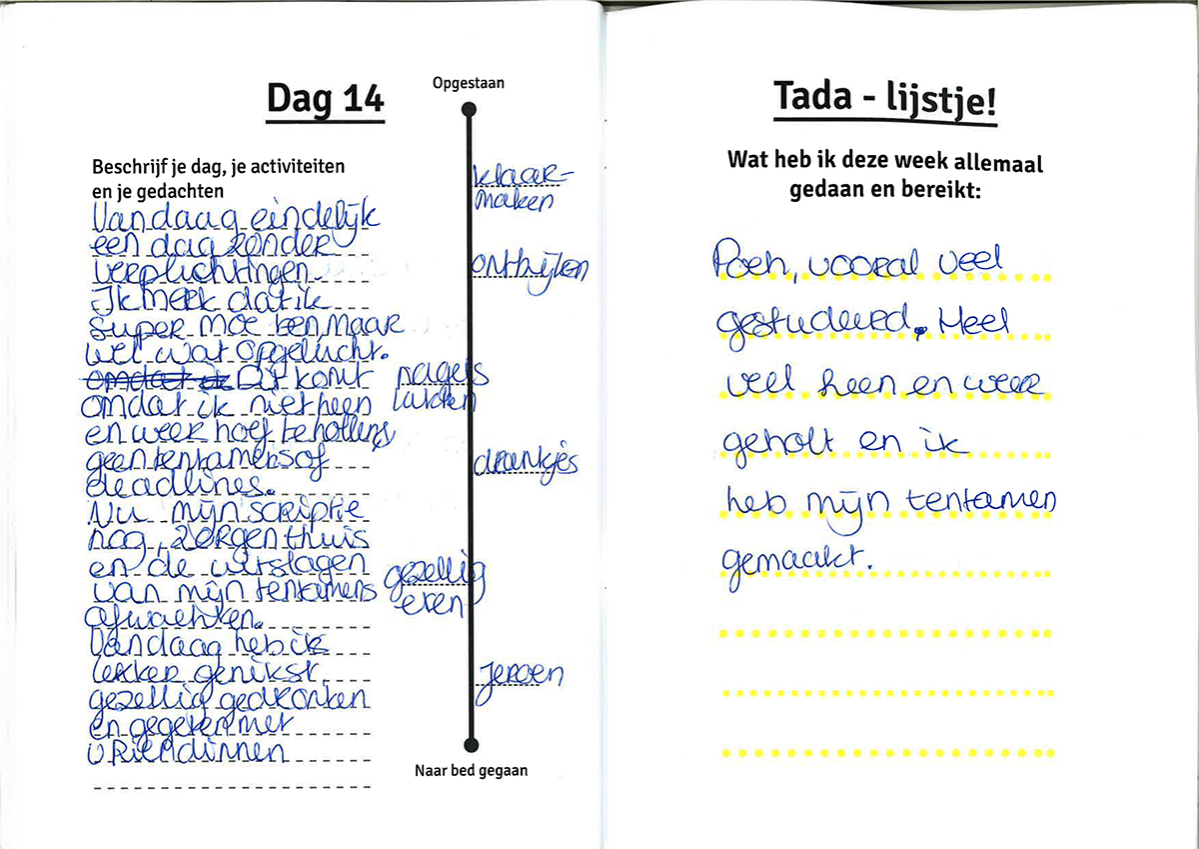
Journal pages from P5. On the left page: a diary of day 14 of the field study and on the right page: a list of things she was proud to have accomplished this week.

**Figure 3:**
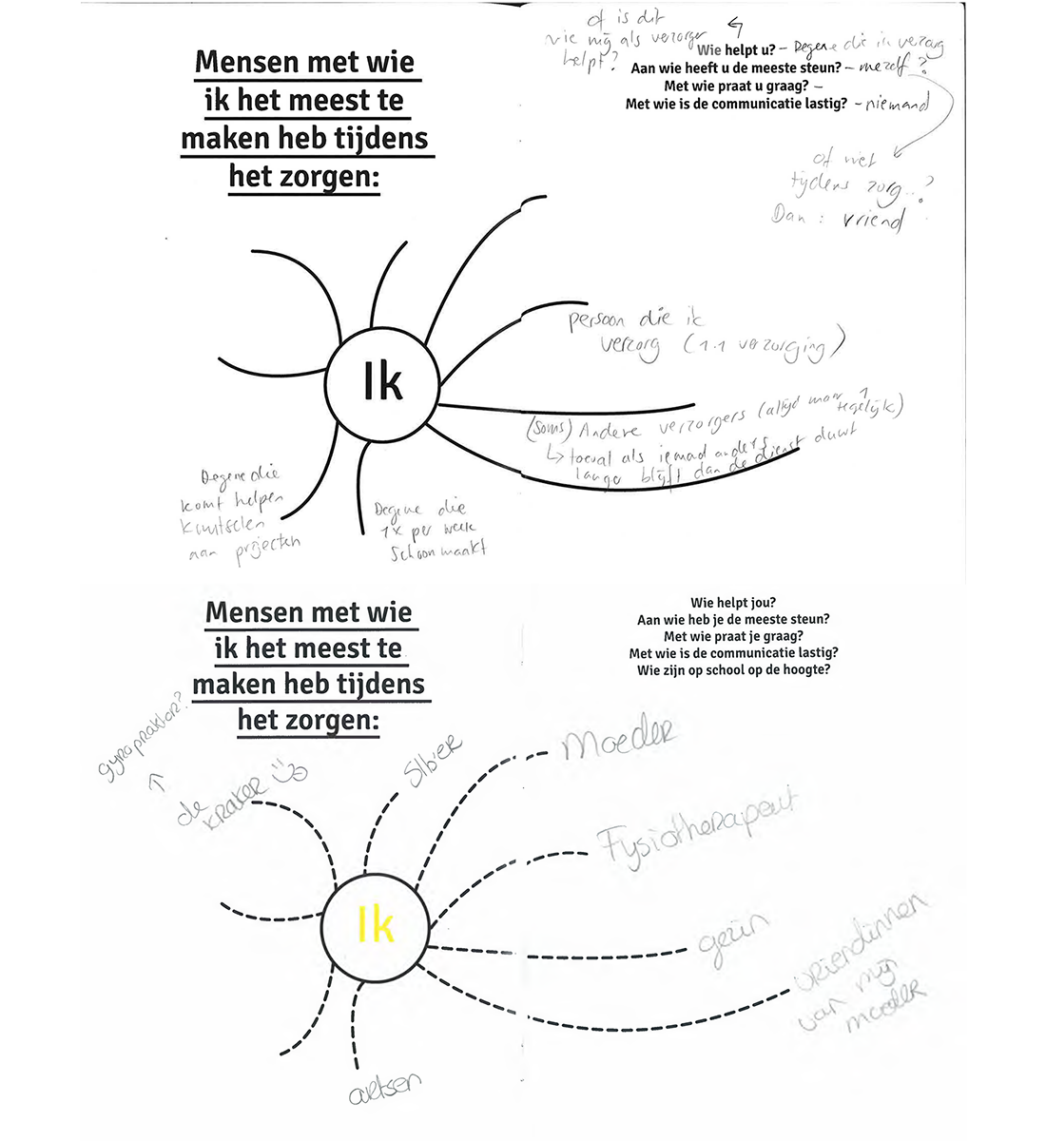
Journal pages from P7 (top) and P4 (bottom). The statement at the top of the page prompts participants: People most involved in the care process. The participant is in the center of the page and writes down who is involved (care recipient, family, friends, and the professional help).

The first tool in the Co-Care-KIT is a custom-designed journal in which the participants can report their activities in their own time during the study. To support this process, the journal includes predesigned pages (see Figures 1-3), for example, some contain a question or a beginning of a mind map about topics such as “positive emotions I am feeling. . .”, “what new skills have you learned from the care you provide?”, or “what do you do to relax?” to provoke reflection about diverse (emotional) experiences; other pages include daily timelines on which participants can note their daily activities.

#### Experience Sampling: Capturing Everyday Moments

**Figure 4:**
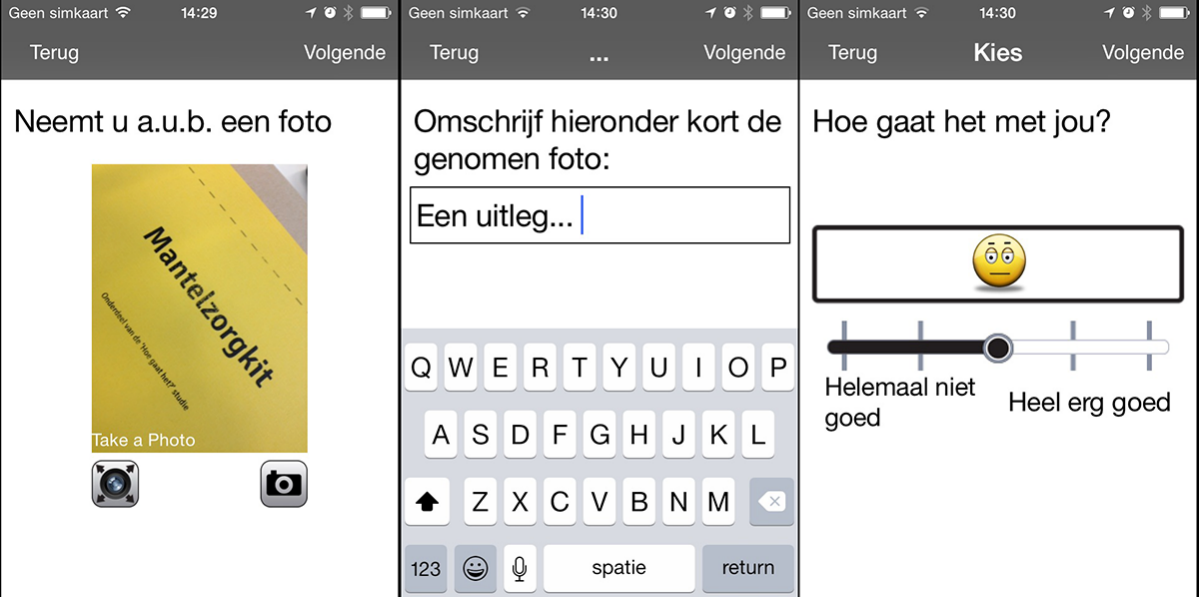
The experience sampling app: taking a picture, writing a caption, and indicating mood.

Second, the Co-Care-KIT contains an iPhone equipped with a photography-based experience sampling app (see Figure 4). The app is programmed to signal participants at random intervals during the day to reflect on what they are doing and how they are feeling. This involves taking a photo and completing a short survey, including writing a caption of the captured moment and indicating how they feel on a 5-point happiness scale. Photo elicitation evokes emotions and feelings and forms a rich source of data. The alarms go off up to a maximum count of 6 times a day so as to capture the experience at different times and showcase a diversity of moments from caregivers over the course of a day. Experience sampling research indicates that a maximum of 6 alarms is considered appropriate to not overburden the participant [57].

Alarms can be set within a time frame in consultation with participating caregivers. Individual alarms can be either snoozed or ignored when these happen to go off at an inconvenient time.

#### Tracking How the Caregiver is Doing

Third, a heart rate monitor wristband (the Mio Link [65]) is included in the Co-Care-KIT to complement the journal entries and photography-based experience sampling data of participants with objective data about how they are doing during the study. This enables caregivers to track their heart rate using an HRV tracking app [66] on the iPhone. HRV is used to describe a number of measurements based on consecutive heartbeats and, in research, HRV measurements are used as a physiological indicator for recognizing mental effort and emotions, such as stress and worry [67-70]. The large body of research done by the MindGames research group [71] showed that biofeedback such as HRV can be creatively used for obtaining a positive state of mind. Current neurobiological evidence suggests that HRV is impacted by stress and supports its use for the objective assessment of psychological health and stress [72]. Nonetheless, measuring physiological data such as HRV with a noninvasive consumer wearable, such as the optical heart rate tracker wristband that was used, is not very reliable. For example, missing a heartbeat because of an incorrectly placed wristband could result in false measurements. However, accuracy was not the main goal but rather to have some type of objective, a physiological indicator to reflect the well-being of caregivers to evaluate if and how caregivers value such data. Therefore, this was nevertheless included in the Co-Care-KIT (with instructions). For this reason, caregivers were able to use the heart rate monitor during the study to track but were not able to see a visual representation of these data until after the field study.

#### Providing Information

Online and offline informative resources for caregivers were included in the kit, as a previous study [8] indicated a clear need for this. These informative materials were obtained and selected through consulting Dutch informal care organizations and included leaflets about support groups for informal caregivers and information about informal caregiver organizations. Moreover, Web page bookmarks with information about informal care support services were preprogrammed on the iPhone.

#### Making Data Discussable

To be able to discuss the collected data samples with caregivers, we see it as critical to visualize each form of experience sample. Therefore, the heart rate measurements of each caregiver were plotted on daily timelines, including the photos that had been taken. This was done to create an overview and to show context data such as time, location, and people. Similarly, the pages in the journal were designed in such a way that these can be interpreted at a glance (see Figures 2 and 3). In summary, the Co-Care-KIT combined various methods for (subjective and objective) experience disclosure into 1 toolkit to enable caregivers to capture and *self-reflect* on their emotional experiences *in situ*. The collected experience samples then served as a rich source for dialogue between participants and researchers.

#### Study Approach

A 2-week field study with the Co-Care-KIT was conducted with home-based informal caregivers in diverse care situations (N=7). This field study was preceded by semistructured introductory interviews with each of the participants to gain an understanding about their general experience of caregiving, and each individual field study concluded with a closing interview (lasting approximately 1.5 hour) during which the data that were collected with the Co-Care-KIT were discussed and evaluated together with the participants.

##### Recruiting Informal Caregivers

The majority of studies with informal caregivers in HCI focus on caregivers of a specific patient group (eg, caregivers of people that are diagnosed with Alzheimer disease). Moreover, even though people of all ages serve as informal caregivers, the majority of studies are focused on adult caregivers [12,54]. This study goes beyond these specific caregiver or patient groups and focuses on capturing the caregiving experiences of diverse caregivers in diverse care situations. An intentionally wide variety of caregivers was approached for recruitment. The challenge in recruiting potential participants is that caregivers often fail to identify with the term *informal caregiver* because of the gradual assumption of the role [73,74]. Therefore, many different approaches were undertaken for recruiting study participants: collaborating with informal care organizations and research groups in the Netherlands and beyond; volunteering as a caregiver to connect with the target population; promotional flyers at a doctors’ office, placing online calls in Facebook caregiver support groups; and reaching out to young caregivers who had already taken part in a university-wide study and who indicated their role and consented to be contacted for future research studies.

##### Study Design

After recruitment and obtaining formal and detailed consent, *semistructured introductory interviews* were conducted with informal caregivers of care recipients with wide-ranging care needs (N=7; see Table 1).

**Table 1:** Overview of study population and collected data.

Participant	Gender	Age	Health concerns of care recipient	Working part-time (PT), full-time (FT), or studying	Relation to care recipient	Hours spent caregiving per week	Duration of field study (days)	Photos collected	Heart rate tracking (days)	Journal kept
P1	M	63	Dementia, osteoporosis, and visual impairment	PT	Son/son-in-law	3-8	8	30	7	Yes
P2	F	36	Stroke, paralysis	PT	Daughter	8-16	14	32	1	Yes
P3	F	35	Spinal cord injury	PT	Neighbor	<3	14	30	11	Yes
P4	F	57	Dementia, osteoporosis	PT	Daughter	8-16	11	42	3	Yes
P5	F	23	Amyotrophic lateral sclerosis	FT student	Daughter	3-8	12	17	1	Yes
P6	F	22	Old age, frailty	FT Student and PT working	Granddaughter	8-16	7	25	3	Yes
P7	F	26	Chronic pain		Daughter	8-16	14	23	5	Yes

**Figure 5:**
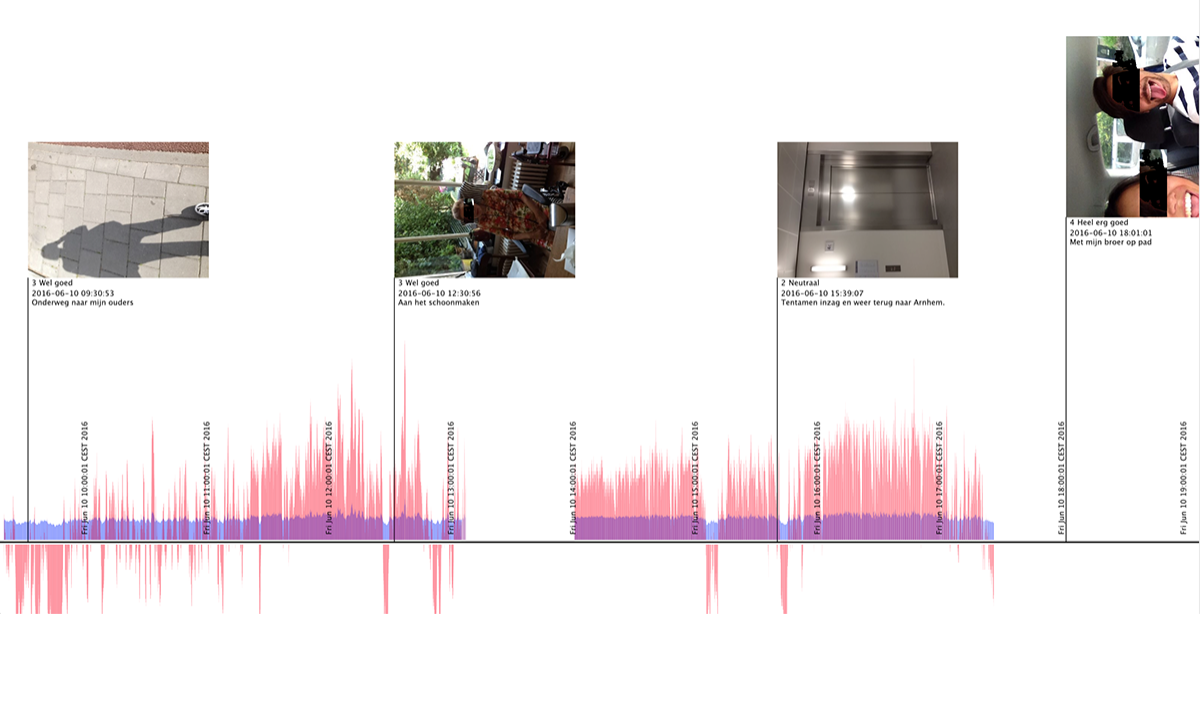
Heart rate measurements of P5 plotted on a 1-day timeline, including the affective state and pictures that were taken that day.

Each caregiver was questioned about (1) *the context of their care situations* (eg, for how long they had been taking care of someone; what their relationship with this person was; why they cared for this person; and what their caregiving tasks entailed) and (2) *their (emotional) experiences of caring for someone* (eg, what they enjoyed most about caregiving and what they did not like about caregiving). An additional theme was discussed with young caregivers who were also enrolled in college, namely (3) *their experience of combining school and caregiving* (eg, how they combined school work, caregiving, and their personal lives; whether their school performance was suffering because of their caregiving work; and how the university could better support them). To gain an understanding of each caregiver’s perceived burden, the 13-question Caregiver Strain Index (CSI) [75] was used during each interview. The CSI is computed by summing the 0 (no) and 1 (yes) responses for the 13 items. Therefore, CSI scores range from 0 to 13. Positive responses to 7 or more items on the index indicate a greater level of strain, and the instrument can be used with individuals of any age. It should be noted that the CSI instrument was originally developed to screen partners of older adults for caregiver strain. For the purpose of this study, a slightly modified version of the CSI was used with all participating caregivers: in one of the questions, the phrase *my partner* was changed to *my care recipient*. Interviews took place in the caregivers’ home or at the university, and each conversation took approximately 1.5 hours including instructions. Owing to the small sample size, the CSI only provides a preliminary insight of participants in the study. Next, a *field study with the Co-Care-KIT study toolkit* followed with all of the participants. As informal caregivers form a study population that can already be quite burdened by their circumstances, the duration of the field study was determined in consultation with each participant and varied between a minimum of 7 days and a maximum of 2 weeks. The informal caregivers were provided with the Co-Care-KIT toolkit and each tool was explained and demonstrated. During the study, the researchers contacted each participant at least once to check if everything was going well and to keep the study motivation going. After the field study was completed, the electronic devices and collected experience data were returned to the researchers and a *final closing interview* was scheduled during which participants discussed and interpreted their captured experience data together with the researchers. In preparation for this interview, participants’ heart rate data were visualized on daily timelines, which also showed the photos that they had taken (Figure 5). These visualizations served as stimulus for the interviews so caregivers could interpret and reflect on their experiences and what they had learned.

##### Data Analysis

All interviews (7 introductory and 7 final interviews) were transcribed and then coded by 2 researchers. The transcriptions were analyzed using applied thematic analysis, a broad inductive method in which key themes are identified in text and transformed into codes [76]. Applied thematic analysis as a method assumes a researcher’s effect on the data. Before the final interviews, the journals that participants had kept during the field study were read and used to formulate additional questions. During the final interviews, the data that were collected with the study kit were thoroughly discussed with each caregiver by using the experience data samples as conversation props: the photos and heart rate measurements were spread out on the table and were discussed and interpreted by the caregivers while the researchers questioned them about it. This was the main method used for analyzing the qualitative data from the kit.

## Results

A group of informal caregivers (N=7, 1 male and 6 females) were recruited for this study (see Table 1). The study population consisted of caregivers aged between 22 and 63 years, amid 3 young caregivers who were still in college.

### Introductory Interviews

The study population included caregivers in diverse care contexts (see Table 1). Most participants cared primarily for their parents (n=5) and in other cases for their grandparents (n=1) or their neighbor (n=1) and cared for persons with diverse and sometimes multiple health issues, varying from Alzheimer disease, old age, and physical disabilities to chronic pain. Overall, caregivers acknowledged a positive outlook on their care situations. Participants seemed very open in discussing their personal experiences during the interviews and the majority of the conversations were perceived optimistic and positive, even while discussing difficult subjects and situations. As P2 explained: “I liked the study method, so that makes it easier to talk about the subject.” However, despite the majority of participants liking the kit and having a seemingly positive outlook and experience of caregiving, their average measured burden was relatively high, namely, 6.7 (SD 2.98) on the CSI. This 13-question instrument is the most used scale for measuring subjective burden among caregivers. The CSI is computed by summing the 0 (no) and 1 (yes) responses for the 13 items. Therefore, CSI scores range from 0 to 13. Positive responses to 7 or more items on the index indicate a greater level of strain. For the adult participating caregivers, the measured CSI was a little lower and more varied, with an average of 6.0 (SD 3.9), than for young caregiver participants, whose average was 7.7 (SD 1.15). Caregivers found it difficult to indicate how much time they actually spent on caregiving on average, as care activities happened routinely throughout the day. Moreover, even though the caregivers had been recruited for a study about the experience of caregivers, they were seemingly trained in thinking about what is best for the person they care for: on multiple occasions during interviews, the researchers had to remind caregivers to answer questions from their own perspectives, instead of from their care recipients’ perspective.

### Field Study With the Co-Care-KIT

The participants used the Co-Care-KIT for 1 to 2 weeks each, with an average of 11 days per participant (see Table 1). Overall, the experience of using the toolkit was found positive and resulted in a considerable amount of experience data recorded by caregivers. In total, 199 photos were captured (mean 28 per participant), 31 days of heart rate measurements were recorded (mean 4.4 days per participant), and every participant used the Co-Care-KIT’s journal during the study. The participants interpreted their captured data together with the researchers during the closing interviews. The findings reported next are based on these interpretations.

### Photography-Based Experience Sampling

Many photos were collected, even though some participants reported that they sometimes skipped recording a sample during the study when consecutive alarms would go off and they were still in the same place or engaging in the same activity that had been recorded before. Signaling participants to capture photos at random moments throughout the day and initiating a small moment of reflection by writing a caption ensured that participants not only captured the highlights of the day but also the *mundane* experiences. This resulted in a varied and detailed visual report of their lives. As P1 stated,

We already take a lot of photos now, but those are the typical “oh look how nice” or “look how special” moments.

Thus, experiences captured with the photo elicitation app were diverse: moments spent in hospital; performing care tasks; during cooking, cleaning, or doing laundry; time spent with family members or friends; at work; at school; during homework; or while traveling. The photos taken during the study revealed a lot about the daily context of each caregiver’s life: their home and the places they visit, their family, the person they care for, and all the different things the caregivers do in a day on top of their care responsibilities.

**Figure 6:**
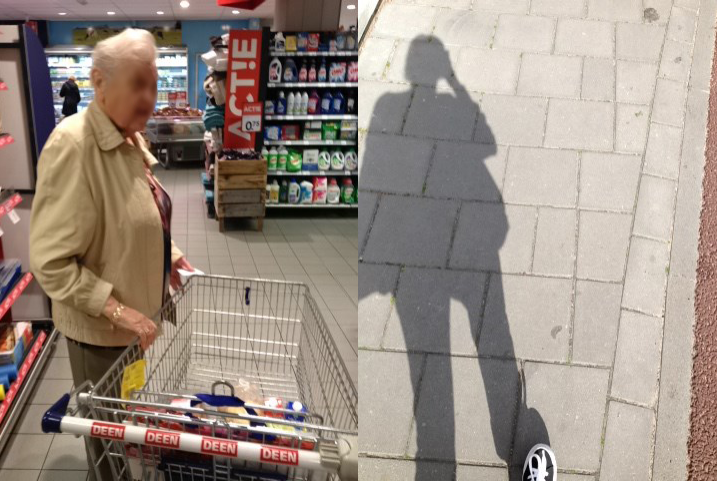
Left: P6 feels appreciated when she accompanies her grandma shopping. Right: P5 feels stressed and irritated when she took this photo: she had a lot of schoolwork to do but instead was on her way to help her parents.

During the closing interviews, each participant was asked to organize and discuss the photos they had captured in whichever way they preferred. The photos that participants most often selected as positive, unsurprisingly, involved activities during leisure time, spending time with friends or family, and cooking. Caregivers also indicated moments spent with the person they cared for as most special to them. Negative moments included times that the caregivers experienced worry or stress: such as during a hospital visit; when the care recipient was not doing well; or, for young caregivers, moments when they were stressing over their homework. Moreover, seeing repetitive photos was confronting for some participants because it emphasized how much time they spent inside the house (P4) or at work (P3). Some participants did not like performing care tasks, whereas others enjoyed them because it made them feel appreciated and helpful. For example, P6 (see Figure 6) stated:

My grandmother is usually really happy when I help her with her groceryshopping. She knows everyone, including me, has a lot on our plates, so when I take my time with her and don’t rush her through the store she is really appreciative and that makes me feel good about it.

Another participant (P5) described how some days were stressful when she had a lot of studying to do, but also had to help her parents with chores (Figure 6). However, there were also photos of moments that were not simply positive or negative and talking about these moments revealed some conflicting feelings a caregiver can experience that were not captured in the photo caption, emotion scale, or journal. For example, 1 caregiver (P6) photographed a sports game that she went to in the weekend and although she had categorized this moment under *relaxing activities*, she explained that during the moment she had felt guilty over not being able to visit her grandfather that day, which made it difficult for her to enjoy the moment. This example also demonstrates how caregivers, even when they are not actively carrying out care activities can still feel emotionally burdened.

Despite the mixed emotions, the photo sampling seemed to help with positive reflection, as P5 stated,

Most valuable was that I had to write a short comment about the photo. I liked that. From the photos I learned that I like where I am and I like what I do, during caregiving.

### Journal Keeping

All of the participants wrote in the journal throughout the study, although with varying detail. They found it was less immediate for capturing experiences than the photo elicitation app. For example, P1 stated,

The journal is less “in the moment”. I wrote in it afterwards, based on memory, not during the day.

Of them, 1 participant (P5) explained it was sometimes hard to be honest in her journal entries on days where she did not accomplish the things that she had planned because she was too tired. Nonetheless, she kept a detailed diary throughout the field study (Figure 2) and was generally positive about it, stating:

I liked the journal a lot. I particularly noticed I liked to fill in the exercises and questions, apart from writing down what I did that day. It made me realize what happens and who are important to me. Writing down my days made me realize how much I accomplish in a day.

The diary provoked personal reflection about how caregiving made them feel and why and about patterns in their own behavior. For example, P7 stated that rereading how hectic her previous day was made her realize why she was so tired at the moment. This helped her to decide she should have a night off. Thus, participants also indicated that the journal made them see all the different things they had accomplished in a day. This supported appreciation of the nice moments they experienced. Especially, the themed pages in the journal were helpful. They helped seeing and appreciating what was important and to look at things with a different perspective, such as identifying small upsides in stressful situations. P7 indicated that the page about people involved in the care process (see Figure 3, top) helped her see that there were more people she could ask for help than she realized. P4 used the same pages in the journal to reflect on the quality in the communication with the people involved (Figure 3, bottom).

### Heart Rate Measurements

Tracking physiological data as an indication of how participants were feeling when they wore them induced some interesting findings. Except for one, all participants found the idea of measuring their physiological well-being interesting, especially for measuring how they felt during specific moments in which they expected an abnormal heart rate (eg, during stressful times, during caregiving, or after exercise). For example, P5 stated,

In the beginning I thought about taking pictures during certain moments, especially in combination with the heart rate measurements. Because I thought it would be interesting to be able to see how it affected me.

Remarkably, the heart rate band got an extra meaning: it became a visual expression of being a caregiver. During the previous interviews, many caregivers explained they did not consciously think of themselves as *informal caregivers* and they often did not talk to their social network about their caregiver status. During the study, however, when a colleague or friends asked a participant about why he/she was wearing a heart rate band, it became a positive cue to talk about their caregiving situations with someone they would normally not.

I’m never very explicit about being a caregiver, but I noticed that when I was doing this study, that people automatically asked me about it. Because I was taking photos and wearing the heart rate wristband. Before, I always thought: Those are my private things. But people found it special that I was making photos and wearing the band. That made it easy to talk about it and people liked that I was doing it. They said: “Oh, I didn’t even know that you were a caregiver”. It felt good to be able to tell them, to be honest.

P2

For the final interview, the heart rate measurements that were recorded during the study were plotted on a daily timeline that also showed the photos that the participant had taken each day (see Figure 5). This combination proved helpful in remembering the context and details of a certain moment and helped put days with negative experiences into perspective as it showed other more positive experiences on the same day as well. For example, when discussing her photos, P5 picked out a photo where she stated that she was very stressed and irritated (Figure 6). However, when the same photo came up in the heart rate plot (Figure 5) she remembered that she ended that same day on a very positive note together with her brother. This was something she had forgotten all about.

There was 1 case with negative feedback in relation to the heart rate wristband. Owing to a technical error, the wrist band’s battery indication light started blinking unexpectedly and this discouraged a participant (P2) to take any more measurements during the field study because it worried her that something was wrong with her heart rate. Overall, the visual representation of their well-being from the heart rate measurements was found reassuring by most of the participants. For example, P4 said,

I am relieved that the measurements don’t show anything too surprising. Sometimes I’m afraid that it gets to me more than I think. It’s reassuring.

### A Deeper Understanding of Caregivers’ Experiences

Through the study, caregivers in our study became (more) aware of their experience of caregiving. A number of dynamic conditions seemed to affect the caregivers particularly in their emotional states and well-being:

- *How the care recipient is doing:* Caregivers expressed that their personal happiness often depended on how the person they cared for was doing and that they were frequently concerned about their loved one, especially when they were not easily able to check in. For example, 1 caregiver (P1) lived far away from his father, making it difficult for him to be there: “I worry a lot about my father, especially because he lives so far away.” Moreover, participants indicated that moments of worry arose around critical moments for the care recipient, for example, during hospital appointments; when waiting on test results; or when hearing about bad outcomes that influence the treatment of the person they care for. This emotional dependence was also found in previous research [8] but the samples from the kit (eg, the photos) made this more visible.
- *Uncertainties and unawareness:* Especially before the field study, caregivers were often unaware of the time they spent caregiving and they were more focused on the health of the person they cared for than their own. After the field study, however, they were able to better indicate how their care responsibilities also affected them in moments that they were previously unaware (eg, feeling guilty about being at a sports game instead of with the care recipient). Moreover, having a visual overview (as with the journal entries or the heart rate visualizations) helped caregivers to see the bigger picture and reevaluate even negative experiences. Moreover, caregivers reported how capturing how they felt over time helped reassure them that they were doing well. It also made them more aware of their role. P6 stated: “I liked the exercises in between the journal entries. They make you think about how you experience caregiving, which things I like, about the future, etc. It makes you more conscious.”
- *Increased responsibility:* Older caregivers discussed how they had to adapt their work schedules, for example, by working less hours. Younger caregivers reported about falling behind on schoolwork or even obtaining severe study delay because of the extent of their care tasks. It was difficult for them to study at home when they experienced emotional burden as an effect of their loved one being ill. There was a clear need expressed for support on this level.
- *Talking about the care role and responsibilities to others:* In general, participants did not talk about their experiences and emotions regarding caregiving with friends much and they were more likely to talk about it with their partner or with other caregivers (if at all). The reasons mentioned for this were as follows: not feeling the need to talk about it with people to whom it did not concern or not wanting to bring it up because it was difficult to tell outsiders. P6 stated: “I just always think it’s hard to tell people I am a caregiver; it has such a negative connotation...” Motivations for caregivers to bring up their caregiving situation to their surroundings were more likely to be out of practical nature than social, such as informing their professional environment when they needed flexibility from work responsibilities. Interestingly, an unexpected outcome of using the heart rate band during the field study was that it seemed to produce a positive angle for caregivers to speak about their care role and responsibilities with people with whom they normally would not. P4 also added: “Participating in the study made me talk to my husband more about the situation.”

### Evaluation of the Study Toolkit

Despite the fact that caregivers are a hard-to-reach user group for study participation because they tend to have little time, the tools in the Co-Care-KIT each lead to the recording of rich data of their experience of caregiving. In comparison with the interviews conducted before the field study, the Co-Care-KIT revealed a deeper understanding of the caregivers’ experiences. Much effort was put into learning about the personal multifaceted experiences during the introductory interviews. However, mainly the highlights of caregiving experiences were discussed. Although participants reported that the study was provoking at times, it also increased awareness of their own well-being and the full extent of their caregiving experience filled with stressors, as well as positive experiences. Caregivers also became more aware of the time spent caregiving. P1, for example, explained,

I learned how to name/recognize certain activities. From the kit I learned that I spend WAY more time caregiving than I thought.

This insight could have lasting benefits, as P1 consequently stated: “I am going to push harder to get more formal help.” Especially because caregivers tend to prioritize the health and well-being of the person they care for over their own, this was an important outcome. Furthermore, capturing experiences contributed to an ongoing reflection and empowered participants to open up about their caregiver status to their social network.

Out of all the tools in the Co-Care-KIT, the most positive reactions concerned using the photo elicitation app. Participants found that taking a photo with the app was a convenient method for capturing experiences. Taking a photo and indicating how they felt through the app supported reflection about how they were doing several times a day. P6 stated,

It made me more aware of what I do. It makes you chew over certain moments: what I was doing or what I did.

Several participants indicated that this small repetitive reflection had a positive effect during the study, for example, P3 stated: “It was reassuring that I realized that I feel very stable.” and P1 said,

Taking a photo makes for an evaluation moment. It makes clear that you are doing something, part of a process. That motivates me in tracking the other aspects of that process as well.

Talking about the moments captured in photos with participants helped researchers better understand the personal experience of caregivers and revealed conflicting feelings that caregivers can experience. Similarly, the journal was also helpful in evoking personal reflection about how caregiving made participants feel.

A particular strength of using the toolkit seemed to lie in the richness of the collected samples and how these made it possible to discuss them. The kit allowed caregivers to record many different data samples during only a 2-week field study. Combining various sources of samples in a visual overview, such as the visualizations that combined heart rate measurements and photos on a daily timeline (see Figure 5), was a helpful prompt during the final interviews because of the contextual details it showed. Discussing these with participants helped in creating a bigger picture of the caregivers’ individual experiences. It helped caregivers put things into perspective, instead of focusing on one moment in particular. This was not only useful for gaining a deeper understanding of caregiver experiences for the researchers. Caregivers were positive about this aspect of the study as well, as it was also insightful to them. The idea of measuring their well-being and health was found interesting by the majority of participants, especially as a reassurance during specific moments that they suspected would affect them (eg, during stressful moments, during caregiving, or after exercise). Finally, the participants seemed to like the reflecting of the positive brought about by the kit, as P1 stated,

The kit helped to focus more on the positive, instead of thinking about the practical things you did that day (eg, cleaning), you remember the feeling you had better.

## Discussion

### Principal Findings

The developments in the field of HCI, in which researchers (inspired by positive psychology) have begun to explore and define how technology can foster users’ well-being [16,19,20], call for new approaches for understanding users’ emotional experiences. This paper builds on those developments by exploring how technology can help capture users’ changing emotional states *in situ* during a 2-week field study. Several aspects of the Co-Care-KIT study method particularly showed promise in supporting caregivers through (1) experience capturing, (2) collaborative reflection, (3) providing reassurance, and (4) social sharing:

1. *Support capturing the bigger (positive) picture:* Solely capturing experiences repeatedly throughout the field study gave participants an opportunity to reflect on their emotional experience (in accordance with Isaacs et al [35]). This ongoing reflection promoted a focus on the bigger picture, instead of only the (positive or negative) highlights of a situation. Furthermore, consistent with other research [50,52,64], the study found that writing, listing accomplishments, and reflecting on specific topics had positively affected participants’ awareness of positive aspects in life. Of all the methods, participants generally enjoyed the photo experience sampling tool the most. This method took little effort and, thus, could provide interesting perspectives to deploy in further longer studies to capture and react to changes in wellness.
2. *Enabling collaborative reflection:* The Co-Care-KIT was found to be a valuable tool for discussing users’ multifaceted experiences. The combination of allowing users to self-report and encouraging reflection resulted in a better articulation of their needs. Visualizing the collected data samples made it possible to then analyze experiences in collaboration with participants. The outcome of this collaborative reflection was not only relevant to the researchers but also an insightful process for participants. Mainly in the focus on this collaborative analysis, the Co-Care-KIT toolkit approach differs from similar research methods such as cultural probes.
3. *Justly reassure users of their health and well-being to increase their confidence in the ability to handle the situation:* There was early evidence that providing reassurance about the caregivers’ health promoted confidence in their emotional ability to handle the situation. Given that many factors can affect the well-being of caregivers over time, this is an interesting finding that deserves further investigation. Overall, focusing on emphasizing and visualizing positive aspects of behavior can indeed promote self-confidence and mental resilience, which are important to caregivers [33].
4. *Provide tools for social sharing:* Wearing the heart rate band provoked discussion among caregivers with their social network about their caregiving status. Moreover, the purpose of being a part of the study empowered the caregivers to do so from a positive perspective. The study produced this unexpected positive side effect, as caregivers often tend to keep their experience to themselves. This highlights that providing empowering tools and positive cues can stimulate not only the awareness of the caregivers’ emotional experience to caregivers, but also to others surrounding them.

### Limitations and Future Work

Caregivers form a difficult-to-reach user group for study participation, as they are already heavily taxed. In concurrence with other studies [6,8], challenges came up in recruiting participants for the more extensive field study, resulting in a small sample size. Participants were easier to recruit in the initial questionnaire study [8] than for the more time-consuming study with the toolkit. The study also seemed to attract participants who were less burdened. The recruitment was most effective at including young caregivers as participants. It has to be noted that the sample is not fully representative for the group of informal caregivers, for example, in terms of male and female participants.

Furthermore, according to an extensive study [77], a large percentage of caregivers are interested in technology, but only few are using it. Thus, this still needs to be addressed in real-world settings. Besides, the Co-Care-KIT was used in a short field study. The aim was to investigate how different methods for capturing experiences each helped gain insight into the experience of caregivers. The themes identified in the applied thematic analysis of the qualitative data may have been affected by a researcher effect in which the data collected, and interpretations generated were partly informed by the investigators’ perceptions [76].

Although it was beyond the scope of this study, it could be interesting to study the emotional states of caregivers over time by, for example, using one of the demonstrated tools for tracking experiences in a longer study (eg, long-term reflection on life events [35]). Furthermore, the field study lasted for 2 weeks, whereas caregivers often serve in a long-term situation. Thus, this study provides only a limited insight into changing experiences and in measuring the impact on emotional experiences and well-being. Even so, the method itself, particularly the journal and photo experience sampling tool, showed promise in giving caregivers greater awareness of their own well-being and encouraged social sharing and could become part of a solution. The study also showed an explicit need for more lightweight approaches and solutions that relieve the burden of caregivers. These should further respond to the changing emotional day-to-day experiences of caregivers in a positive way.

### Conclusions

There is a vast opportunity for HCI to better understand and support informal caregivers’ emotional wellness during caregiving through technology. This study showed the relevance of studying the emotional experience of informal caregivers and demonstrated a method for capturing those rich experiences *in situ* with a reflective toolkit. The toolkit was evaluated during a 2-week field study. This study found that through using the Co-Care-KIT method, participants became better able to articulate how their caregiving experiences influenced their well-being. The toolkit method enabled a collaborative analysis together with the participants about both the stressors (such as worrying, changes in their relationship with the care recipient, and having to adapt to the increased responsibilities that come with the caregiver role) as well as the positive experiences of caregivers (such as moments of gratitude, talking about caregiving with their social network, and finding reassurance in handling the situation). This study contributes to a better understanding of the day-to-day experiences of caregivers, which is a first step toward designing solutions for supporting the diverse and changing needs of caregivers at the right time and in the right context.

## Abbreviations

CSI: Caregiver Strain Index
ESM: experience sampling method
HCI: human-computer interaction
HRV: heart rate variability
